# Silicon reduces cadmium accumulation by suppressing expression of transporter genes involved in cadmium uptake and translocation in rice

**DOI:** 10.1093/jxb/erx364

**Published:** 2017-10-14

**Authors:** Ji Feng Shao, Jing Che, Naoki Yamaji, Ren Fang Shen, Jian Feng Ma

**Affiliations:** 1 State Key Laboratory of Soil and Sustainable Agriculture, Institute of Soil Science, Chinese Academy of Sciences, Nanjing, China; 2 Institute of Plant Science and Resources, Okayama University, Chuo, Kurashiki, Japan

**Keywords:** Cd accumulation, down-regulation, OsNramp5, OsHMA2, rice, silicon, toxicity

## Abstract

Silicon (Si) alleviates cadmium (Cd) toxicity and accumulation in a number of plant species, but the exact molecular mechanisms responsible for this effect are still poorly understood. Here, we investigated the effect of Si on Cd toxicity and accumulation in rice (*Oryza sativa*) by using two mutants (*lsi1* and *lsi2*) defective in Si uptake and their wild types (WTs). Root elongation was decreased with increasing external Cd concentrations in both WTs and mutants, but Si did not show an alleviative effect on Cd toxicity in all lines. By contrast, the Cd concentration in both the shoots and roots was decreased by Si in the WTs, but not in the mutants. Furthermore, Si supply resulted in a decreased Cd concentration in the root cell sap and xylem sap in the WTs, but not in the mutants. Pre-treatment with Si also decreased Cd accumulation in the WTs, but not in the mutants. Silicon slightly decreased Cd accumulation in the cell wall of the roots. The expression level of *OsNramp5* and *OsHMA2* was down-regulated by Si in the WTs, but not in the mutants. These results indicate that the Si-decreased Cd accumulation was caused by down-regulating transporter genes involved in Cd uptake and translocation in rice.

## Teaser

In rice Si decreases Cd accumulation in both the roots and shoots by suppressing the expression of transporter genes for Cd uptake and translocation.

## Introduction

Cadmium (Cd) is a heavy metal highly toxic for all organisms in its ionic form. In plants, Cd toxicity has been associated with leaf chlorosis, growth inhibition and the disruption of key physiological processes such as photosynthesis (Atal *et al.*, 1991; Chugh and Sawhney, 1999; Clemens and Ma, 2016). Consumption of foods containing Cd is potentially health-threatening in humans. It was reported that exposure to Cd causes many diseases such as cancers of the prostate and lungs, and kidney malfunction (Nawrot *et al.*, 2006). For example, itai-itai disease was caused by the consumption of rice grown in cadmium-polluted soils in Japan in the mid-1950s and mid-1960s (Horiguchi *et al.*, 1994). Therefore, limiting the entry of Cd into the food chain from the soil is important for reducing potential health risks to humans. This is especially important for rice as it is a staple food for nearly half of the world’s population and the largest source of dietary intake of Cd (Watanabe *et al.*, 2004; Cheng *et al.*, 2006).

Many studies have been carried out on the molecular mechanisms and practical control of Cd accumulation in rice. Several of the transporters involved have been identified (Clemens and Ma, 2016). For example, OsNramp5 (*Oryza sativa* natural resistance-associated macrophage protein 5) was found to be a major transporter for Cd uptake in rice roots (Ishimaru *et al.*, 2012; Sasaki *et al.*, 2012). It is polarly localized at the plasma membrane of both exodermis and endodermis in the roots and responsible for the transport of Cd from the soil solution to the root cells. On the other hand, OsHMA3 (*Oryza sativa* heavy metal ATPase 3) is a tonoplast-localized transporter for Cd, which is involved in the sequestration of Cd into the vacuoles of root cells (Ueno *et al.*, 2010; Miyadate *et al.*, 2011). A homolog of OsHMA3, OsHMA2, however, is localized to the plasma membrane of pericycle cells of the roots and is involved in the root-to-shoot translocation of Cd (Satoh-Nagasawa *et al.*, 2012; Takahashi *et al.*, 2012; Yamaji *et al.*, 2013). At reproductive growth stage, OsHMA2 and OsLCT1 (*Oryza sativa* low-affinity cation transporter 1) were implicated in the distribution of Cd to the rice grain (Uraguchi *et al.*, 2011; Yamaji *et al.*, 2013).

Silicon (Si) has been reported to decrease Cd toxicity and accumulation in rice, which is a typical Si-accumulating species (Wu *et al.*, 2013). For example, supplying Si decreased the concentration of Cd in rice shoots by 24% (Shi *et al.* 2005). Application of calcium silicate significantly reduced the Cd concentration in rice straw and grain (Wang *et al.*, 2016). Several possible mechanisms for this Si-induced decrease of Cd accumulation have been proposed including (i) decreased apoplastic transport of Cd due to Si deposition in the cell wall of the endodermis and epidermis (Shi *et al.*, 2005), (ii) the formation of a [Si–hemicellulose matrix]Cd complex and subsequent co-precipitation (Liu *et al.*, 2013; Ma *et al.*, 2015), (iii) down-regulation of genes involved in Cd accumulation (Kim *et al.*, 2014; Ma *et al.*, 2015), and (iv) decreased soil Cd availability due to pH increase after silicate fertilizer application (Liang *et al.*, 2005). However, the exact molecular mechanism for the Si-induced decrease of Cd toxicity and accumulation is still poorly understood.

In the present study, we used two mutants defective in Si uptake and performed physiological and molecular comparison with their wild types on the interaction between Si and Cd in rice. These two mutants have a large difference in shoot Si accumulation, but have similar Si accumulation in the roots compared with their respective wild types (Ma *et al.*, 2002, 2006, 2007). Therefore, these mutants enable us to separate the effect of Si in the shoots and roots on Cd accumulation and toxicity. We found that Si does not have a direct effect on alleviating Cd toxicity in rice but that Si decreases Cd accumulation by down-regulating transporter genes involved in uptake and root-to-shoot translocation of Cd. Furthermore, we found that Si bound to the cell wall of the roots does not contribute to Si-decreased Cd accumulation.

## Materials and methods

### Plant materials and growth conditions

Two rice (*Oryza sativa*) mutants (*lsi1* and *lsi2*) and their wild types (cvs Oochikara and T-65) were used in this study. The rice mutants, *lsi1* and *lsi2*, have a point mutation in the Si influx transporter (Lsi1) and efflux transporter (Lsi2), respectively (Ma *et al.*, 2006, 2007). Seeds were soaked in water for 2 d at 30 ºC in the dark and then the geminated seeds were transferred to nylon nets ﬂoating on a solution containing 0.5 mM CaCl_2_ in a 1.2-liter pot and grown for 5 d. The seedlings were then transferred to a 3.5-liter pot containing a 1/2 Kimura B nutrient solution (pH 5.6). The solution contained the following macronutrients (mM): MgSO_4_ (0.28), (NH_4_)_2_SO_4_ (0.18), Ca(NO_3_)_2_ (0.18), KNO_3_ (0.09), and KH_2_PO_4_ (0.09); and micronutrients (µM): Fe-EDTA (20), H_3_BO_3_ (3), MnCl_2_ (0.5), CuSO_4_ (0.2), ZnSO_4_ (0.4), and (NH_4_)_6_Mo_7_O_24_ (1); it was renewed every 2 d. All experiments were repeated at least three times with three to four replications each in a greenhouse under natural sunlight at 20–30 ºC. To obtain a similar size of plants for different experiments, we used seedlings with different ages.

### Effect of Si on Cd-induced inhibition of root elongation

To investigate the effect of Si on Cd toxicity in rice, 5-day-old seedlings were exposed to a 0.5 mM CaCl_2_ solution (pH 5.1) containing various Cd concentrations (0, 0.5, 1, 2, and 5 µM) for 24 h with or without 1 mM Si (as silicic acid). Silicic acid was prepared by passing potassium silicate through a cation exchange resin (Amberlite IR-120B) (Ma *et al.*, 2002). Root length was measured by a ruler before and after the Cd treatment.

### Effect of Si on Cd accumulation in roots and shoots

The effect of Si on Cd accumulation was investigated by exposing the seedlings (17 d old) to a nutrient solution containing 1 µM Cd in the presence and absence of 1 mM Si as silicic acid. The treatment solution was renewed every 2 d. At 7 day after the exposure, the roots were washed with 5 mM cold CaCl_2_ three times and separated from the shoots. Both the roots and shoots were subjected to determination of Cd as described below.

To determine time-dependent effect of Si on Cd accumulation, seedlings (22 d old) of both mutants and the wild types as prepared above were transferred to a 1.2-liter pot (four plants per pot) and cultured for a further 9 d. The seedlings were subsequently exposed to a nutrient solution containing 1 µM Cd with or without 1 mM Si for different durations (1, 3, 5, and 9 d). The roots and shoots exposed for different times were harvested as described above at the same day.

### Effect of different Si concentration on Cd accumulation

Seedlings (22 d old) of both mutants and the wild types were exposed to a nutrient solution containing 1 µM Cd in the presence of different Si concentrations (0, 0.2, 0.5, 1, and 2 mM). The treatment solution was renewed every 2 d. After exposure for 7 d, the roots and shoots were separately harvested as described above.

### Effect of Si pre-treatment on Cd accumulation

To examine the effect of pre-treatment with Si on Cd accumulation, seedlings (15 d old) of both mutants and the wild types were first cultivated in the nutrient solution with or without 1 mM Si for 7 d. These seedlings were subsequently exposed to 1 µM Cd in the absence or presence of 1 mM Si, respectively. After 24 h, the roots and shoots were harvested as described above.

### Cd concentration in root cell sap and xylem sap

For collection of xylem sap, seedlings (11 d old) were exposed to a nutrient solution containing 1 µM Cd for 7 d without or with 1 mM Si, and the shoot (2 cm above the root) was excised with a razor; then the xylem sap was collected with a micropipette for 1 h after decapitation of the shoot. The Cd concentration was determined by inductively coupled plasma mass spectrometry (ICP-MS) as described below.

Seedlings (17 d old) of both mutants and the wild types were cultivated in a nutrient solution containing 0 or 1 mM Si for 7 d. Subsequently, these seedlings were exposed to a nutrient solution containing 1 μM Cd with or without 1 mM Si. After 24 h, the roots were washed with cold 5 mM CaCl_2_ three times and placed on a filter in a tube and then frozen at −80 °C overnight. For collection of root cell sap, the sample was thawed at room temperature, followed by centrifugation at 20600 *g* for 10 min.

### Effect of Si on Cd binding to root cell wall

Root cell wall was prepared by boiling roots of seedlings (18 d old) pre-treated with or without 1 mM Si for 7 d in methanol for 5 min. The roots were then washed three times with fresh methanol, followed by distilled water three times. The roots were blotted with paper and exposed to a 20 ml solution containing 50 µM CdSO_4_ (for a sufficient amount of Cd adsorption) and 0.5 mM CaCl_2_ in a 50 ml plastic tube. The tube was shaken occasionally. At 5, 10, 30, 60, and 120 min, an aliquot of 50 µl was sampled for Cd determination as described below. At the end of experiment, the root cell wall was washed three times in cold 0.5 mM CaCl_2_ and dried in an oven. The dried sample was subjected to digestion as described below.

### Determination of metals in plant tissues

The samples harvested were dried at 70 °C in an oven for 3 d. Digestion was conducted with concentrated HNO_3_ (60%) at a temperature up to 140 °C. The metal concentration in the digested solution, xylem sap, root cell sap and treatment solution was determined by ICP-MS (7700X; Agilent Technologies) after appropriate dilution.

### Expression analysis of Cd transporter genes

To examine the effect of Si on the expression level of *OsHMA2*, *OsHMA3*, and *OsNramp5* in the mutants and the wild types, seedlings (10 d old) were cultivated in a nutrient solution containing 0 or 1 mM Si. After 7 d, the seedlings were exposed to 0 or 1 μM Cd in the presence or absence of 1 mM Si for another 24 h. The roots were then harvested and frozen in liquid nitrogen. Total RNA was extracted with an RNeasy Plant Mini Kit (Qiagen). After the reaction with DNase I (Invitrogen, http://www.invitrogen.com/), 0.5 µg of total RNA was used for first-strand cDNA synthesis using a SuperScript II kit (Toyobo) following the manufacturer’s instructions. The expression of *OsHMA2*, *OsHMA3*, or *OsNramp5* was determined with SsoFast EvaGreen Supermix (Bio-Rad) on a quantitative RT-PCR machine (CFX384; Bio-Rad). Primers used were 5′-CATAGTGAAGCTGCCTGAGATC-3′ and 5′-GATCAAACGCATAGCAGCATCG-3′ for *OsHMA2*; 5′-TCCATCCAACCAAACCCGGAAA-3′ and 5′-TGCCAA TGTCCTTCTGTTCCCA-3′ for *OsHMA3*; 5′-CAGCAGCAGTAA GAGCAAGATG-3′ and 5′-GTGCTCAGGAAGTACATGTTGA T-3′ for *OsNramp5*. *HistoneH3* was used as an internal standard with primer pairs 5′-AGTTTGGTCGCTC TCGATTTCG-3′ and 5′-TCAACAAGTTGACCACGTCACG-3′. The relative expression was normalized by the ΔΔ*C*_t_ method using CFX Manager software (Bio-Rad).

### Split root experiment

To examine the effect of Si accumulation in the shoots on the expression of *OsHMA2*, *OsHMA3*, and *OsNramp5* in the roots, a split root experiment was carried out according to Mitani-Ueno *et al.* (2016). Roots of rice seedlings (18 d old, cv. Oochikara) were split into two parts. Half roots were exposed to 360 ml of 1/2 Kimura B solution without Si (–Si) in a plastic container (left), while the other half roots were exposed to the same solution but containing 1 mM Si in a separate container (right), designed as –Si+Si. As controls, split roots were exposed to –Si or +Si in both compartments, designed as –Si–Si or +Si+Si. The treatment solutions were renewed every 2 d. The Si concentration in the solution of separate compartments was determined daily and no Si was detected in the –Si compartment. After 1 week, the roots in different compartments were exposed to 1 µM Cd in the presence or absence of Si. After 24 h, the roots were separately harvested for RNA extraction as described above. The expression of *OsHMA2*, *OsHMA3*, and *OsNramp5* was determined by quantitative RT-PCR as described above.

### Immunostaining of roots

An antibody against OsNramp5 used in the previous study was used for immunostaining of OsNramp5 (Sasaki *et al.*, 2012). The synthetic peptide MEIERESSERGSISWRASA-C (positions 1–19 of OsNramp5) was used to immunize rabbits to obtain antibodies against OsNramp5. The obtained antiserum was puriﬁed through a peptide afﬁnity column before use. Roots (19-day-old seedlings) of two mutants and the wild types pre-treated with 1 mM Si or not for 7 d were sectioned and used for the immunostaining as described previously (Yamaji *et al.*, 2008). Fluorescence of the secondary antibody (Alexa Fluor 555 goat anti-rabbit IgG; Molecular Probes) was observed with a confocal laser scanning microscope (TCS SP8x; Leica Microsystems) under the same conditions.

### Statistical analysis

Significance of differences between means was assessed by Duncan’s test.

## Results

### Effect of Si on Cd-induced inhibition of root elongation

**Fig. 1.**
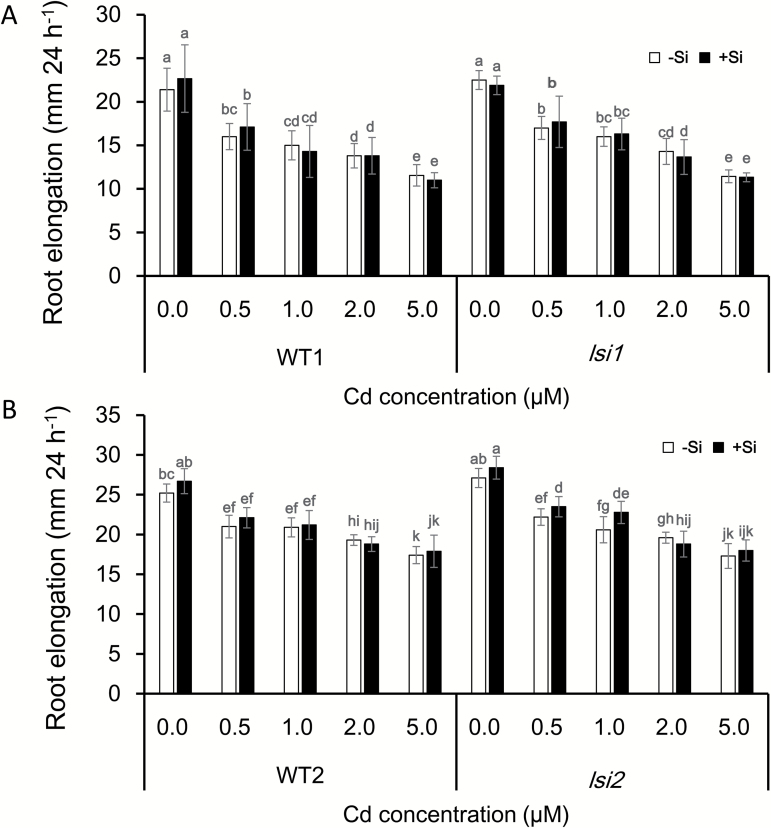
Effect of Si on Cd-induced inhibition of root elongation. Seedlings (5 d old) of *lsi1*, *lsi2*, and their wild types (WT1 for *lsi1* and WT2 for *lsi2*) were exposed to different Cd concentrations for 24 h. The root length was measured by a ruler before and after exposure to Cd. Data are means±SD (*n*=10). Different lower-case letter indicates significant difference at *P*<0.05 by Duncan’s test.

Root elongation is the most sensitive indicator of metal toxicity (Barcelo and Poschenrieder, 2002). We compared root elongation during 24 h between roots with and without Si in the presence of different Cd concentrations. The root elongation decreased with increasing Cd concentrations in external solution in all lines (Fig. 1). However, there was no difference in the Cd-inhibited root elongation between roots with or without Si. There was also no difference in the root elongation between *lsi1* and its wild type, or *lsi2* and its wild type at either Cd concentration (Fig. 1). These results indicate that Si does not have direct alleviative effect on Cd toxicity.

### Si decreased Cd accumulation in wild types, but not in mutants

**Fig. 2.**
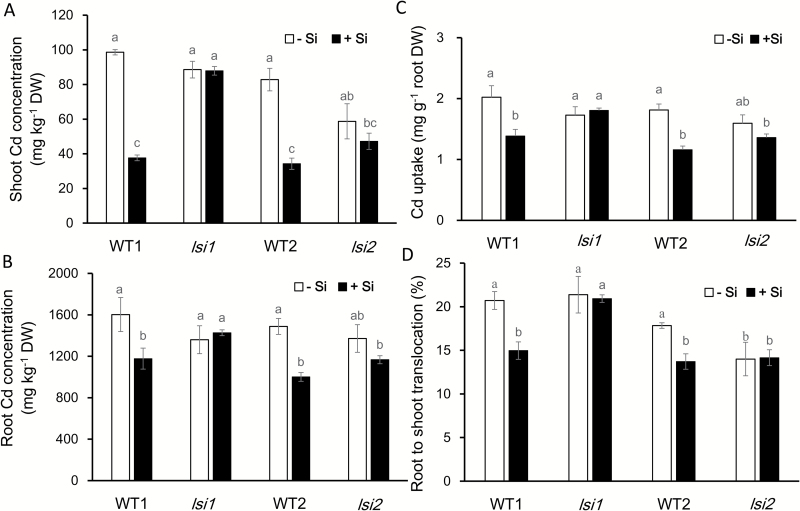
Effect of Si on Cd accumulation, uptake and translocation in mutants and their wild types. (A, B) Cd concentration in shoots (A) and roots (B). (C) Cd uptake. (D) Root-to-shoot translocation of Cd. Seedlings (17 d old) of *lsi1*, *lsi2*, and their wild types (WT1 for *lsi1* and WT2 for *lsi2*) were exposed to a nutrient solution containing 1 µM Cd with or without 1 mM Si for 7 d. Roots and shoots were harvested for determination of Cd concentration. Data are means±SD (*n*=3). Different lower-case letter indicates significant difference at *P*<0.05 by Duncan’s test.

Addition of Si as silicic acid for 7 d significantly decreased Cd concentration in both the shoots and roots of the two wild types (Fig. 2A, B). However, such a decrease was not found in the two mutants. The total Cd uptake was decreased by Si in the two wild types, but not changed in both the *lsi1* and *lsi2* mutants (Fig. 2C). Root-to-shoot translocation of Cd was also decreased by Si in the wild types, but not altered in the mutants (Fig. 2D).

**Fig. 3.**
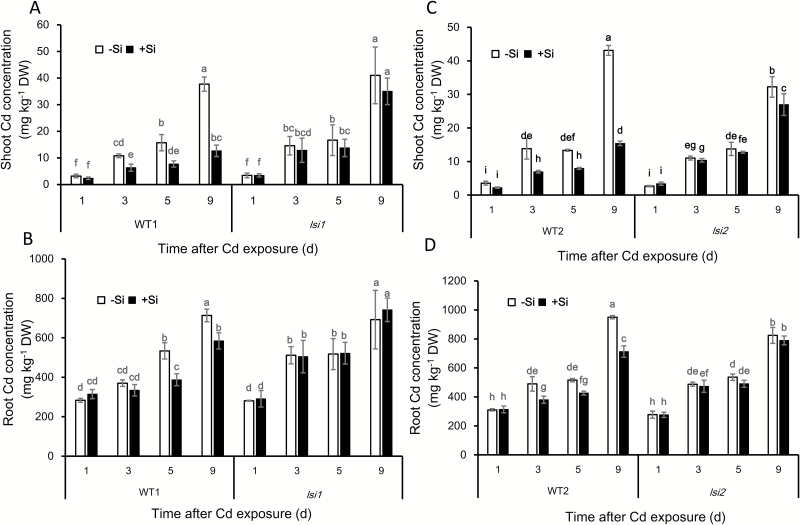
Time-dependent effect of Si on Cd accumulation in shoots and roots. (A, B) Cd concentration in the shoots (A) and roots (B) of *lsi1* and its wild type (WT1); (C, D) Cd concentration in the shoots (C) and roots (D) of *lsi2* and its wild type (WT2). Seedlings (22 d old) of *lsi1*, *lsi2*, and their wild types were exposed to a nutrient solution containing 1 µM Cd with or without 1 mM Si for 1, 3, 5, and 9 d. Roots and shoots were harvested for determination of Cd concentration by ICP-MS. Data are means±SD (*n*=4). Different lower-case letters indicate significant difference at *P*<0.05 by Duncan’s test.

A time-course experiment showed that Si-decreased Cd concentration could be found at day 3 after addition of Si in both roots and shoots of the wild types (Fig. 3). The difference in shoot Cd concentration became evident with prolonged time of Si addition. Compared with the Cd in the roots, the difference in the shoot Cd concentration was larger between plants with Si and without Si in both wild types. No difference in Cd concentration of both roots and shoots was found in the two mutants at all times sampled (Fig. 3).

### Effect of different Si concentrations on Cd accumulation

Shoot Cd concentration decreased with increasing Si concentrations (up to 2 mM) in the solution in the two wild types (see Supplementary Fig. S1A at *JXB* online). However, the shoot Cd concentration was unaffected by either Si concentration in the two mutants. The root Cd concentration showed similar trends to the shoots although the effect of Si was not as large as that in the shoot (Fig. S1B).

### Effect of Si pre-treatment on Cd accumulation in roots and shoots

**Fig. 4.**
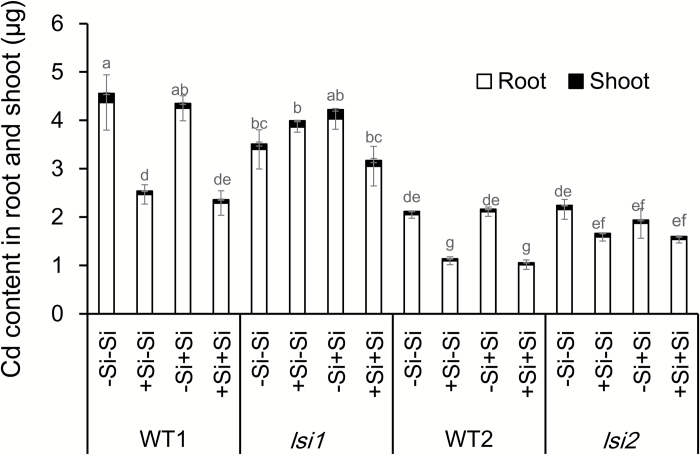
Effect of Si pre-treatment on Cd accumulation in *lsi1*, *lsi2*, and their wild types (WT1 for *lsi1* and WT2 for *lsi2*). Seedlings (15 d old) pre-treated with or without 1 mM Si for 7 d were exposed to 1 µM Cd in the presence or absence of 1 mM Si for another 24 h. Shoots and roots were harvested for determination of Cd concentration and total accumulation was calculated based on biomass and Cd concentration. Data are means±SD (*n*=3). Different lower-case letters indicate significant difference at *P*<0.05 by Duncan’s test.

To investigate pre-treatment with Si on Cd accumulation, we exposed seedlings pre-treated with Si or not to 1 µM Cd in the presence or absence of 1 mM Si for 24 h. In the wild types, pre-treatment with Si significantly decreased Cd accumulation (–Si–Si *vs* +Si–Si) (Fig. 4). One day exposure with Si did not affect the Cd accumulation (–Si–Si *vs* –Si+Si). There was no difference in the Cd accumulation between plants with +Si–Si and +Si+Si (Fig. 4), indicating the residual effect of Si accumulated in the plants. By contrast, in the two mutants, no difference in the Cd accumulation was found between plants pre-treated with or without Si (Fig. 4).

### Si decreased Cd concentration in xylem sap and root cell sap in wild types, but not in mutants

Cd concentration in the xylem sap was compared between plants cultivated in the absence and presence of Si. In the two wild types, plants with Si showed significantly lower Cd concentration in the xylem sap compared with those without Si treatment (see Supplementary Fig. S2A). However, in the two mutants, the Cd concentration in the xylem sap was similar between plants with and without Si treatment.

Cd concentration in the root cell sap was also compared. The Cd concentration in the root cell sap was lower in plants with Si pre-treatment than those without Si pre-treatment in the two wild types (Supplementary Fig. S2B). By contrast, the Cd concentration in the root cell sap was similar in the two mutants with or without Si pre-treatment (Supplementary Fig. S2B).

### Effect of Si on Cd binding to the root cell walls

**Fig. 5.**
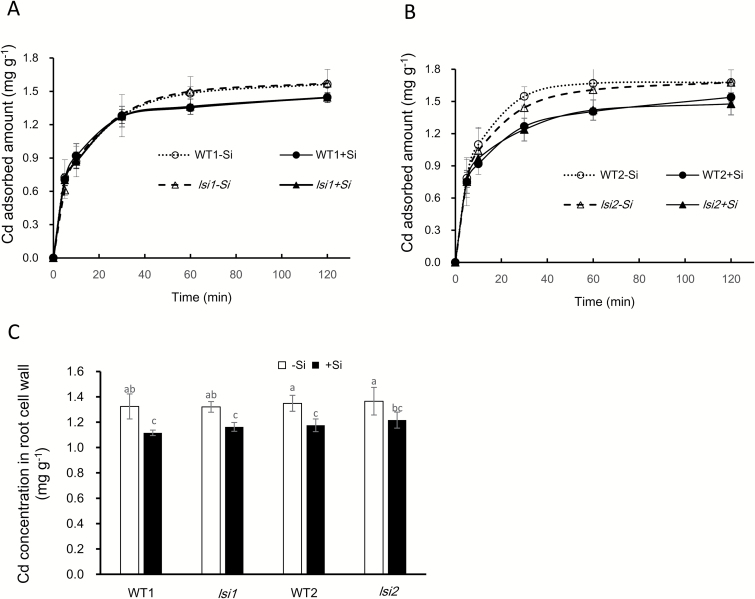
Effect of Si on Cd binding to the root cell wall. (A, B) Time-dependent binding of Cd to the cell wall of *lsi1* and its wild type (WT1) (A), and *lsi2* and its wild type (WT2) (B). (C) Cd concentration in root cell wall of *lsi1*, *lsi2*, and their wild types (WT1 and WT2). Root cell wall was prepared by boiling in methanol roots of seedlings (18 d old) pre-treated with or without 1 mM Si for 7 d. The Cd binding experiment was conducted by incubating the cell wall in a 0.5 mM CaCl_2_ solution containing 50 µM Cd. An aliquot of 50 µl solution was sampled at different times up to 120 min. At the end of experiment, the root cell wall was collected and subjected to Cd determination by ICP-MS. Data are means±SD (*n*=3). Different lower-case letters indicate significant difference at *P*<0.05 by Duncan’s test.

To examine the effect of Si on Cd binding to the cell wall, we prepared the cell wall of the roots pre-treated with Si or not. A time-course experiment showed that Cd binding to the cell wall increased with time till 60 min after exposure to Cd and reached saturation thereafter in all lines (Fig. 5A, B). There was no difference in the Cd binding to the cell wall between roots with and without Si until 30 min in all lines (Fig. 5A, B). However, at 60 min and thereafter, Cd binding to the cell wall was slightly lower in the roots with Si than those without Si (Fig. 5A, B). Analysis with the root cell wall after the exposure also showed that Cd binding to the cell wall was slightly lower (10–15%) in the roots with Si than those without Si (Fig. 5C).

### Effect of Si on the expression of *OsNramp5*, *OsHMA2*, and *OsHMA3*

**Fig. 6.**
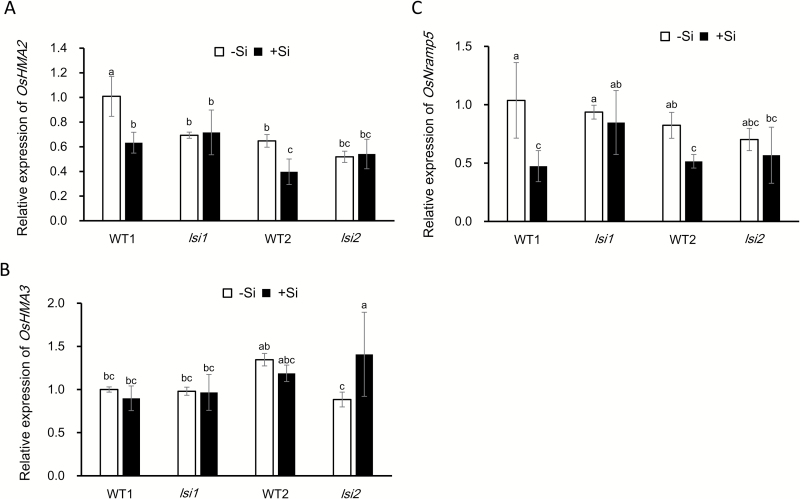
Effect of Si on the expression of *OsHMA2*, *OsHMA3*, and *OsNramp5* in roots of *lsi1*, *lsi2*, and their wild types (WT1 for *lsi1* and WT2 for *lsi2*). Seedlings (10 d old) were cultivated in a solution with or without 1 mM Si for 7 d. Before sampling for RNA extraction, the roots were exposed to 1 µM Cd for 24 h. The expression of *OsHMA2* (A), *OsHMA3* (B), and *OsNramp5* (C) was determined by quantitative RT-PCR. *HistoneH3* was used as an internal standard. Expression relative to WT (–Si) is shown. Data represent means±SD (*n*=4). Different lower-case letters indicate significant difference at *P*<0.05 by Duncan’s test.

At vegetative growth stage, three transporters are involved in Cd accumulation, namely OsHMA2, OsHMA3, and OsNramp5 (Clemens and Ma, 2016). We investigated the effect of Si on the expression level of *OsHMA2*, *OsHMA3*, and *OsNramp5* in the two mutants and their wild types with or without Si in the absence and presence of 1 µM Cd. In the two wild types, the expression level of *OsHMA2* and *OsNramp5* in the roots was down-regulated by Si, regardless of the absence or presence of 1 µM Cd (Fig. 6 and Supplementary Fig. S3). By contrast, the expression level of *OsHMA3* was not affected by Si (Fig. 6 and Supplementary Fig. S3). However, Si did not affect the expression level of three genes in the two mutants (Fig. 6).

### Effect of Si accumulated in the shoots on the expression of *OsNramp5*, *OsHMA2*, and *OsHMA3*

**Fig. 7.**
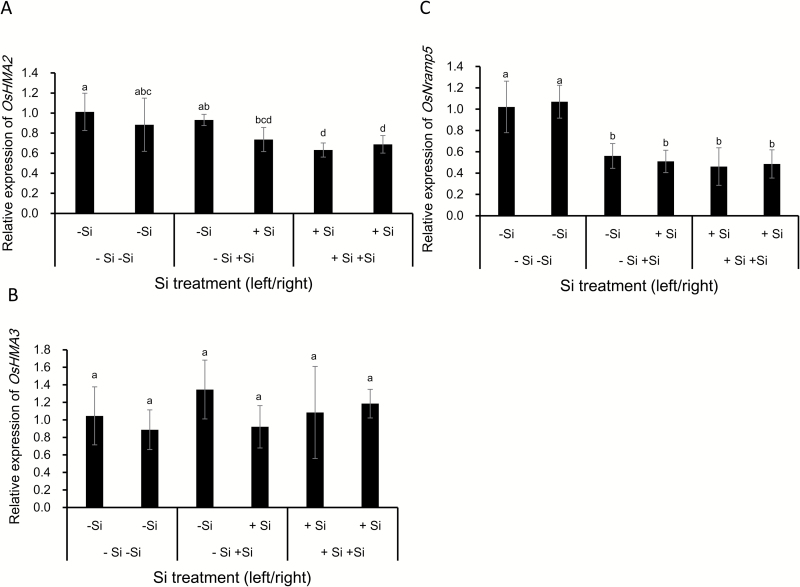
Effect of shoot Si accumulation on expression of *OsHMA2*, *OsHMA3*, and *OsNramp5* in the rice roots. A split root experiment was conducted by splitting the roots of seedlings (18 d old) without Si into two parts. Half roots were exposed to 1 mM Si (+Si) (right) and the other half to 0 mM Si (–Si) (left) in a separate compartment. Split roots exposed to –Si or +Si in both compartments were used as controls. After a 7-d culture, all the roots were exposed to 1 µM Cd for 24 h in the presence or absence of Si as pre-culture and the roots in different compartments were separately sampled for RNA extraction. The expression of *OsHMA2* (A), *OsHMA3* (B), and *OsNramp5* (C) was determined by quantitative RT-PCR. *HistoneH3* was used as an internal standard. Expression relative to –Si roots (left) is shown. Data are the means±SD (*n*=4). Different lower-case letters indicate significant difference at *P*<0.05 by Duncan’s test.

To investigate whether Si accumulation in the shoots is required for down-regulating Cd transporter genes, we performed a split root experiment. Similar to the results shown in Fig. 6, addition of Si in both compartments resulted in reduced expression of *OsNramp5* and *OsHMA2*, but not that of *OsHMA3* by comparing +Si+Si with –Si–Si in split roots (Fig. 7). When half roots were exposed to 1 mM Si, the expression of *OsNramp5* in the other half roots without Si exposure was also down-regulated in the –Si+Si treatment (Fig. 7). However, the expression of *OsHMA2* and *OsHMA3* in the half roots without Si was hardly affected by the other half roots with Si (Fig. 7).

### Effect of Si on OsNramp5 protein

**Fig. 8.**
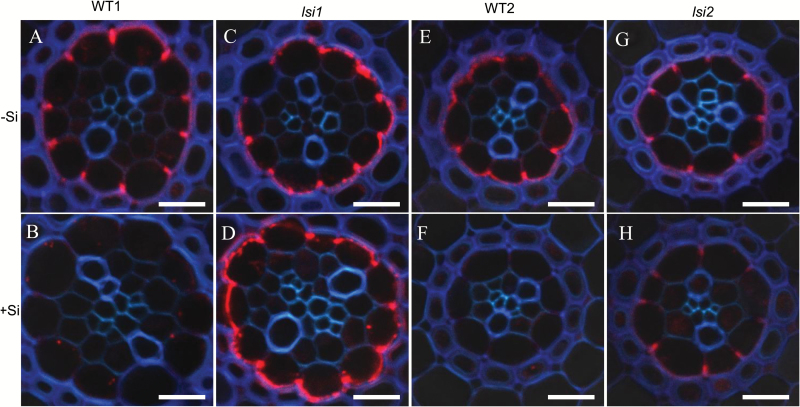
Effect of Si on abundance of OsNramp5 protein in roots. Fine lateral roots of wild type 1 (cv. Oochikara) (A, B), *lsi1* (C, D), wild type 2 (cv. T-65) (E, F), and *lsi2* (G, H) were used for immunostaining. The seedlings were pre-treated without (A, C, E, G) or with (B, D, F, H) Si for 7 d. Bars: 10 µm.

We further investigated whether Si affected the tissue localization and protein level of OsNramp5 in fine lateral roots. Although OsNramp5 was polarly localized at the distal side of endodermis in all lines (Fig. 8), Si pre-treatment reduced the signal of OsNramp5 in the wild type, but not in the two mutants. This result is consistent with the gene expression result (Fig. 6).

## Discussion

Si has various beneficial effects on plant growth by alleviating biotic and abiotic stresses (Ma, 2004; Ma and Yamaji, 2006). One of them is to alleviate metal toxicity (Ma, 2004; Pontigo *et al.*, 2015), but the molecular mechanisms underlying this beneficial effect are poorly understood. In the present study, we took advantage of using two mutants (*lsi1* and *lsi2*) defective in Si uptake and examined the interaction between Cd and Si in rice at both physiological and molecular levels. Rice is a Si-accumulating plant, which is able to accumulate more than 5% Si by dry weight (Ma and Takahashi, 2002). Its high Si accumulation is attributed to the high Si uptake ability of the roots, which is mediated by two different Si transporters, Lsi1 and Lsi2 (Ma *et al.*, 2006, 2007). Lsi1 and Lsi2 function as influx and efflux transporter, respectively. Both transporters are localized at the exodermis and endodermis of the roots, but show different polar localization; Lsi1 is polarly located at the distal side, while Lsi2 is at the proximal side (Ma *et al.*, 2006, 2007). They form a cooperative system for efficient uptake of Si (Ma and Yamaji, 2015). Knockout of either *Lsi1* or *Lsi2* resulted in loss of Si uptake (Ma *et al.*, 2006, 2007). Since more than 90% of Si taken up is immediately translocated to the shoots (Ma and Takahashi, 2002), the mutants of *lsi1* and *lsi2* show large difference in Si accumulation in the shoots, but similar Si accumulation in the roots (Ma *et al.*, 2002, 2007). Therefore, these mutants provide good materials to dissect out the effect of Si on Cd toxicity and accumulation in rice. Our detailed physiological and molecular characterization revealed that Si does not have a direct effect on alleviating Cd toxicity and that Si-induced decrease of Cd accumulation is due to shoot Si accumulation-mediated down-regulation of transporter genes involved in Cd uptake and translocation.

### Si does not alleviate Cd toxicity directly

Many studies have reported that Si was able to alleviate Cd toxicity in different plant species based on comparison of root and shoot biomass between plants with and without Si (Shi *et al.*, 2005; Vaculik *et al.*, 2009; Rizwan *et al.*, 2012; Farooq *et al.*, 2013). However, we found that Si did not alleviate Cd-induced inhibition of root elongation during 24 h in both wild types and the two mutants (Fig. 1). Root elongation is the most sensitive indicator of metal toxicity, which has been used in many studies. The inconsistency probably results from different duration of Cd exposure. In most studies, the plants were exposed to Cd for a long period (Nwugo and Huerta 2008; Zhang *et al.*, 2008; Rizwan *et al.*, 2016). Since Si is able to decrease Cd accumulation (Tripathi *et al.*, 2012) (Fig. 2A, B), this decreased Cd accumulation will improve growth indirectly. Therefore, the alleviative effect of Si observed in previous studies results from Si decreasing accumulation of Cd indirectly. In addition, the concentrations of Cd used differed in different studies. In our experiment, we used low Cd concentrations (up to 5 μM), which are realistic concentrations in soil solution (Mench and Martin 1991; Knight *et al.*, 1997; Degryse *et al.*, 2003). Even at these low concentrations, root elongation was inhibited (Fig. 1). However, in most other studies, the concentration of Cd used was very high (e.g. 50 μM). This high concentration will also cause some other indirect effects. For example, high Cd inhibited the uptake of Mg and K (Rubio *et al.*, 1994), which will also affect the root growth. In addition, that there is no alleviative effect of Si on Cd toxicity also suggests that Si as silicic acid is not able to form a complex with Cd in treatment solutions.

### Si hardly affects binding of Cd to the cell wall

Similar to other studies (Shi *et al.*, 2005; Zhang *et al.*, 2008; Lin *et al.*, 2016), we found that Si effectively reduced Cd accumulation in rice plant, especially in the shoots (Fig. 2A). However, this effect was not observed in the two mutants defective in Si uptake (Fig. 2A). One possible mechanism for this Si-decreased accumulation of Cd was proposed to be formation of a [Si–hemicellulose matrix]Cd complex and subsequent co-deposition in the cell wall (Ma *et al.*, 2015), which inhibits Cd uptake in rice suspension cells. However, this seems not to be the case in the rice roots. We found that Si decreased Cd accumulation in both the roots and shoots (Figs 2A, B and 3), and that the Cd binding to the root cell wall was not increased but rather slightly decreased by Si (Fig. 5). Furthermore, there was no difference in Cd binding of the cell wall between the wild types and mutants although a large difference in shoot Cd accumulation was found (Figs 2A and 5C). These results clearly indicate that Cd binding to the cell wall is not responsible for the Si-decreased accumulation of Cd in rice. This is supported by a recent study that reported no Cd–Si co-localization in the roots of durum wheat (Rizwan *et al.*, 2016). A fraction of Cd taken up by the roots will be translocated to the shoots in plants, but in suspension cells there is no such root-to-shoot translocation process, and this is probably responsible for the different results observed in roots and suspension cells. In addition, the cell wall properties may differ between root cells and suspension cells (Fischer *et al.*, 1996).

### Down-regulation of Cd transporter genes is responsible for Si-decreased accumulation of Cd

At the vegetative growth stage, Cd accumulation is at least controlled by three transporter genes: *OsNramp5*, *OsHMA2*, and *OsHMA3*. Uptake of Cd by the roots is mediated by OsNramp5 in rice (Sasaki *et al.*, 2012). OsNramp5 transports both Mn and Cd. Our previous study showed that Si decreased Mn uptake by down-regulating *OsNramp5* expression in a wild-type rice (cv. Oochikara) (Che *et al.*, 2016). In the present study, we confirmed this result in another wild type rice (cv. T-65) (Fig. 6C). This result is also in agreement with other studies although the expression pattern is somewhat different (Ma *et al.*, 2015, 2017). Interestingly, the expression level of *OsNramp5* was unaffected by Si in both *lsi1* and *lsi2* mutants (Fig. 6C). These results indicate that down-regulation of *OsNramp5* by Si contributes to decreased Cd accumulation in the wild type rice. This is in agreement with physiological data; the concentration of Cd in the root cell sap was decreased by Si in the wild types but not in mutants (see Supplementary Fig. S2B). Furthermore, although Si did not alter tissue localization of OsNramp5, the protein level was decreased in the wild types, but not in the mutants (Fig. 8). These results are somewhat different from that of Lin *et al.* (2017). They found that *OsNramp5* was down-regulated in WT, *Lsi1*-overexpressed lines and RNAi lines in the absence of Cd.

In the present study, we also found that the expression of *OsHMA2* was down-regulated by Si in the wild types, but not in the two mutants. OsHMA2 is localized at the root pericycle cells and is responsible for the translocation of Cd and Zn from the roots to the shoots in rice (Yamaji *et al.* 2013). Knockout of *OsHMA2* results in decreased root-to-shoot translocation of Cd in rice. Therefore, in addition to decreased Cd uptake by Si, down-regulation of *OsHMA2* is also responsible for the Si-decreased accumulation of Cd in the shoots. This is supported by lower root-to-shoot translocation of Cd in the wild types, but not in the mutants (Fig. 2D). Although Si also decreases the root-to-shoot translocation of Mn in rice (Che *et al.*, 2016), the mechanism seems different. In case of Mn, Si is proposed to form a complex with Mn in the root cells, resulting in increased Mn in the root cell sap (Che *et al.*, 2016). By contrast, Si decreased Cd concentration in the root cell sap (see Supplementary Fig. S2B).

OsHMA3 is a tonoplast-localized transporter for Cd in rice roots (Ueno *et al.*, 2010). It is responsible for sequestration of Cd into the vacuoles. Therefore, loss of function of this gene will increase Cd accumulation in the shoots. Different from *OsNramp5* and *OsHMA2*, we found that Si did not affect the expression of *OsHMA3* (Fig. 6B). This result is different from those of Kim *et al.* (2014) and Farooq *et al.* (2016), who found that Si down-regulated expression of *OsHMA3*. However, their results cannot explain the phenotype of Si-decreased accumulation of Cd. One possible reason is that the Cd concentration used in their studies was too high (e.g. 100 µM) so that the root function was seriously damaged.

### High Si accumulation in the shoots is required for down-regulating Cd transporter genes

Si-induced down-regulation of *OsNramp5* and *OsHMA2* expression was only observed in the two wild types of rice, not in the two mutants (Fig. 6A, C), suggesting that high Si accumulation in the shoots is required for this down-regulation because mutants have similar Si content in the roots to the wild types (Ma *et al.*, 2002, 2007). This is supported by the time-course and dose–response experiments. A clear Si-induced decrease of Cd accumulation was found at day 3 after Si supply (Fig. 3), suggesting that sufficient accumulation of Si is required. Furthermore, Cd accumulation in the shoots was decreased with increasing Si concentrations in the external solution (see Supplementary Fig. S1). Pre-treatment with Si also resulted in decreased Cd accumulation when exposed to Cd for 1 d in the absence of Si (Fig. 4), whereas only 1 d culture with Si did not affect Cd accumulation. This evidence suggests that some signal such as a small peptide or plant hormone comes from the shoots to suppress the expression of *OsNramp5* and *OsHMA2*. This was supported by the split root experiment, especially for *OsNramp5* (Fig. 7); the expression of *OsNramp5* in the half roots without direct contact with Si was also suppressed when the other half roots were exposed to Si (Fig. 7). However, a similar phenomenon was not observed for *OsHMA2*, suggesting a different mechanism for Si-suppressed expression of *OsHMA2*, although the exact molecule remains to be identified in future.

In conclusion, our results show that Si does not have a direct effect on alleviating Cd toxicity in rice. However, Si is very effective in decreasing Cd accumulation, especially in the shoots. This effect is attributed to down-regulation of *OsNramp5* and *OsHMA2*, which are involved in uptake and root-to-shoot translocation of Cd. Furthermore, high Si accumulation in the shoots is required for this down-regulation.

## Supplementary data

Supplementary data are available at *JXB* online.

Fig. S1. Effect of different Si concentrations on Cd accumulation in rice shoots and roots.

Fig. S2. Effect of Si on Cd concentration in the xylem sap and root cell sap of *lsi1*, *lsi2*, and their wild types (WT1 for *lsi1* and WT2 for *lsi2*).

Fig. S3. Effect of Si on the expression of *OsHMA2*, *OsHMA3*, and *OsNramp5* in roots of *lsi1*, *lsi2*, and their wild types (WT1 for *lsi1* and WT2 for *lsi2*) in the absence of Cd.

## Supplementary Material

Supplementary Figures
