# Supplementary material for: Silicon reduces cadmium accumulation by suppressing expression of transporter genes involved in cadmium uptake and translocation in rice
Source: J Exp Bot. 2017 Oct 14;68(20):5641–51. doi: 10.1093/jxb/erx364 (PMC5853798; doi:10.1093/jxb/erx364)
Supplement: Supplementary Figures [file erx364_suppl_supplementary_figures.pdf]

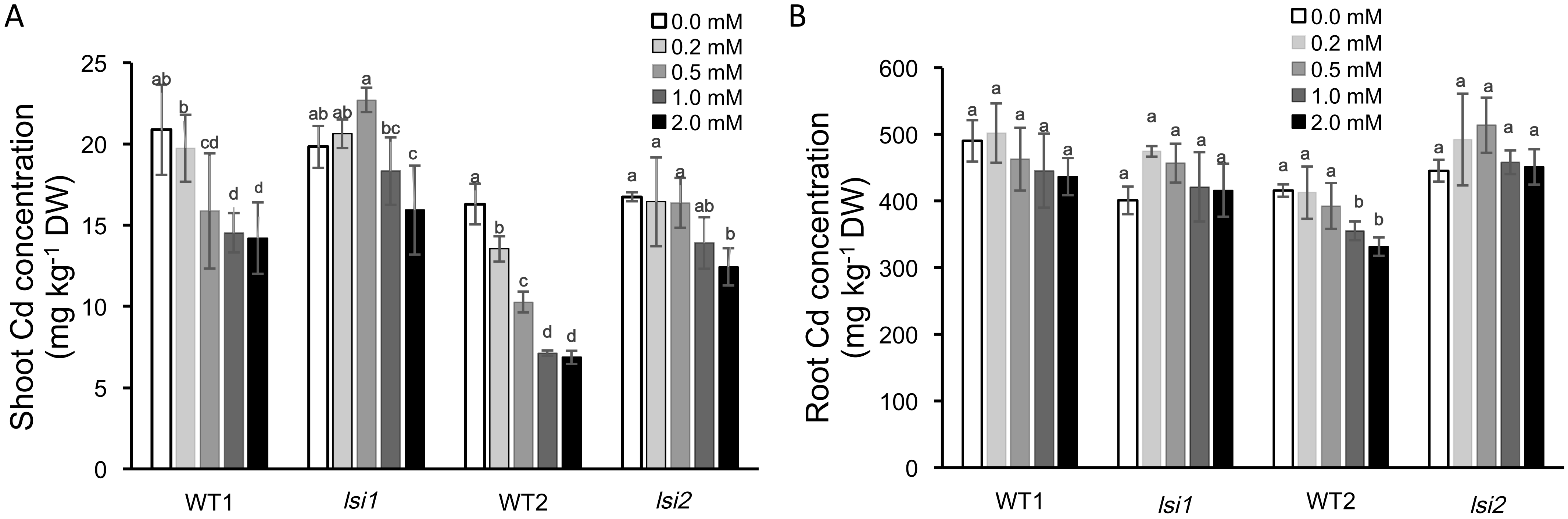


Fig.S1. Effect of different Si concentrations on Cd accumulation in rice shoots and roots. (A-B) Cd concentration in the shoots (A) and roots (B) of *lsi1*, *lsi2* and their wild types (WT1 for *lsi1* and WT2 for *lsi2*). Seedlings (22-d-old) were exposed to a nutrient solution containing 1 μM Cd with different Si concentrations ranging from 0 to 2 mM as silicic acid for 7 d. Shoots and roots were harvested for determination of Cd by ICP-MS. Data are means ± SD (n=3). Different small letter indicates significant difference at $p < 0.05$ by Duncan's test.

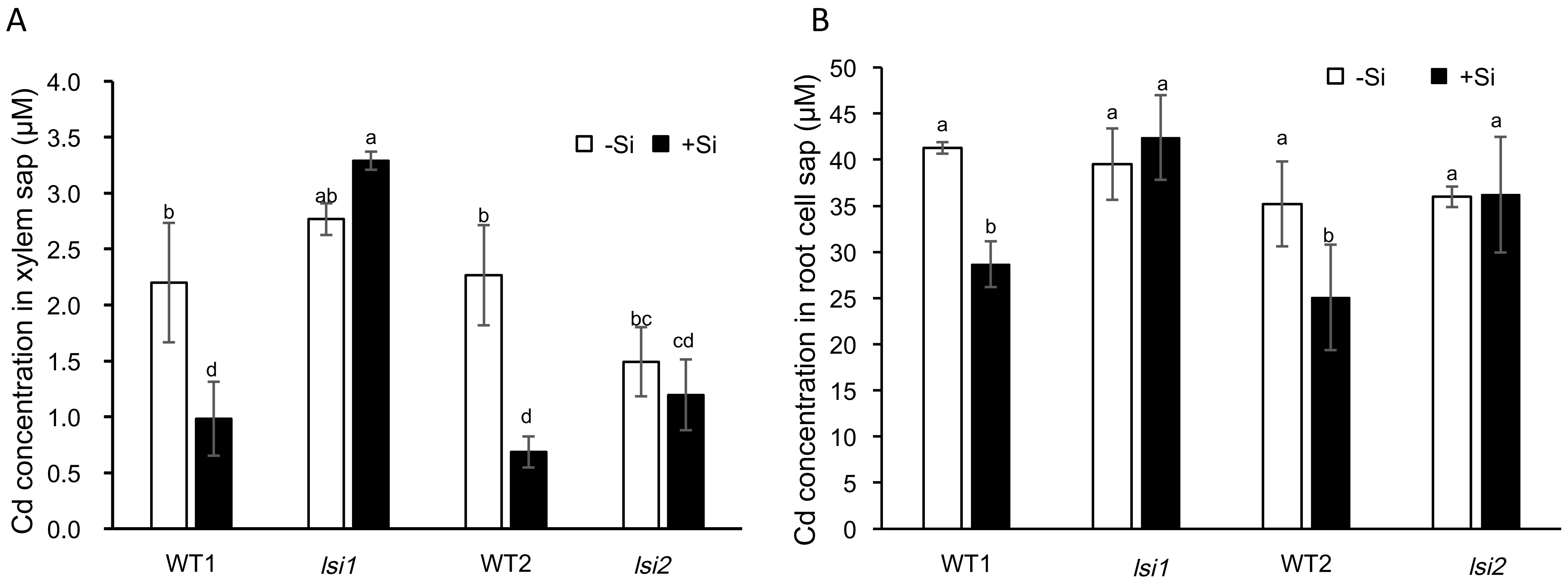


Fig. S2. Effect of Si on Cd concentration in the xylem sap and root cell sap of *lsi1*, *lsi2* and their wild types (WT1 for *lsi1* and WT2 for *lsi2*). (A) Cd concentration in xylem sap. Seedlings (11-d-old) were exposed to a nutrient solution containing 1 μM Cd for 7 d without or with 1 mM Si, and the shoot (2 cm above the root) was excised with a razor, and then the xylem sap was collected with a micropipette for 1 h after decapitation of the shoot. (B) Cd concentration in cell sap. Seedlings (17-d-old) were cultivated in a nutrient solution containing 0 or 1 mM Si for 7 d then exposed to a nutrient solution containing 1 μM Cd with or without 1 mM Si for another 24 h, root cell sap was collected by centrifugation. Data are means ± SD (n = 3). Different small letter indicates significant difference at $p < 0.05$ by Duncan's test.

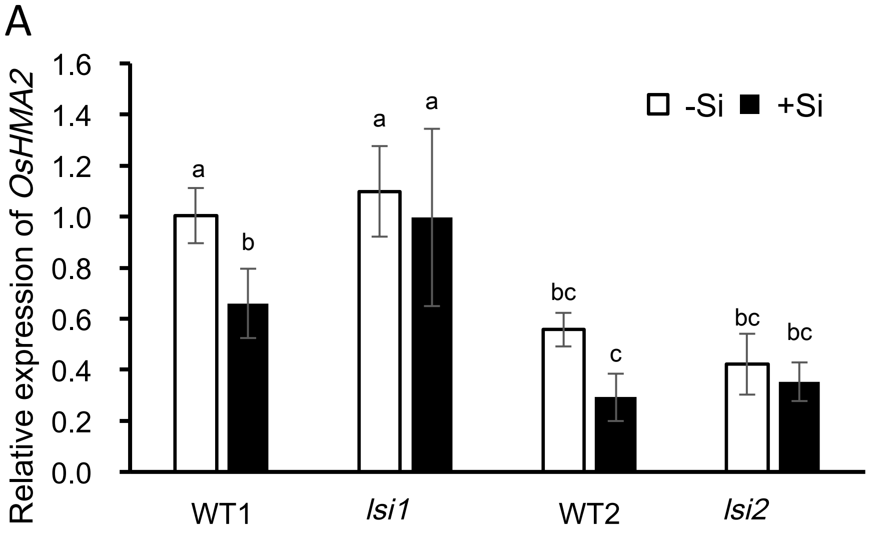


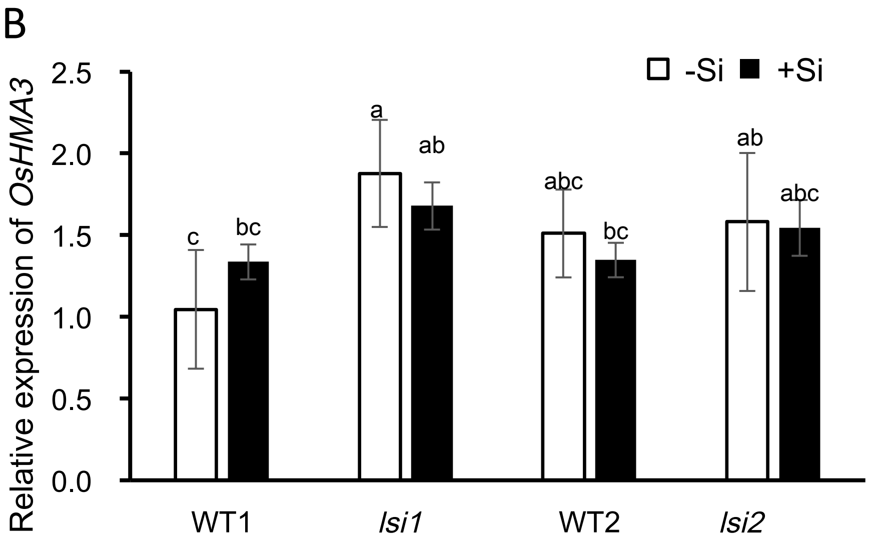


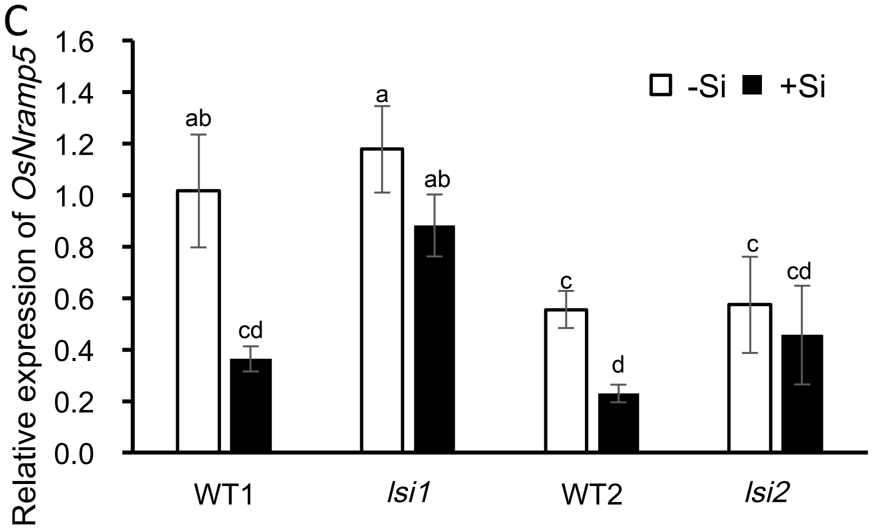


Fig.S3. Effect of Si on the expression of *OsHMA2*, *OsHMA3* and *OsNramp5* in roots of *lsi1*, *lsi2* and their wild types (WT1 for *lsi1* and WT2 for *lsi2*). Seedlings (10-d-old) were cultivated in a solution with or without 1 mM Si for 8 d. The roots were sampled for RNA extraction. The expression of *OsHMA2* (A), *OsHMA3* (B) and *OsNramp5* (C) was determined by quantitative RT-PCR. *HistoneH3* was used as an internal standard. Expression relative to WT (-Si) is shown. Data are means± SD (n=4). Different small letter indicates significant difference at $p < 0.05$ by Duncan's test.
